# Comparative Efficacy of Medical Treatments for Thyroid Eye Disease: A Network Meta-Analysis

**DOI:** 10.1155/2018/7184163

**Published:** 2018-12-12

**Authors:** Nuo Xu, Yi Cui, Tianlu Xie, Mi Zheng

**Affiliations:** ^1^Department of Ophthalmology, Fujian Provincial Hospital, Fuzhou, Fujian, China; ^2^Department of Ophthalmology, Fujian Medical University Union Hospital, Fuzhou, Fujian, China; ^3^Department of Ophthalmology, Fujian Provincial Hospital Southern Branch, Fuzhou, Fujian, China

## Abstract

**Purpose:**

Numerous medical strategies have been proposed for the treatment of thyroid eye disease (TED); however, the best methods for standard treatment are still a matter of controversy. The purpose of this network meta-analysis was to integrate previous evidence to create hierarchies of comparative efficacy of eleven commonly used medical treatments for TED.

**Methods:**

A comprehensive search of electronic scientific literature databases was performed and the data from randomized controlled trials (RCTs) comparing treatment outcomes for patients with active TED were selected. Treatment strategies included in this network meta-analysis were intravenous glucocorticoids (IVGC), oral glucocorticoids (OGC), orbital injection of glucocorticoids (OIGC), orbital radiotherapy (OR), intravenous glucocorticoids combined with orbital radiotherapy (IVGC + OR), oral glucocorticoids combined with orbital radiotherapy (OGC + OR), rituximab (RTX), somatostatin analogs, intravenous immunoglobulin (IVIG), teprotumumab, and cyclosporine. The outcomes were response rate, mean difference in proptosis reduction, and reduction in disease activity. A random-effects network meta-analysis using a frequent method was conducted in STATA.

**Results:**

Twenty-three studies comprising a total of 1047 patients were included in the analysis. Inconsistency plots showed heterogeneity in the IVGC-Placebo-RTX loop to some extent (RoR = 8.029, *P*=0.075). Rankings of response rates were as follows: IVGC + OR, teprotumumab, IVGC, OGC + OR, RTX, OIGC, OR, IVIG, OGC, somatostatin, placebo, and cyclosporine. The rank probability analysis of proptosis reduction showed that teprotumumab was the most effective, followed by IVGC, IVGC + OR, OIGC, OGC, OGC + OR, OR, somatostatin, cyclosporine, and placebo.

**Conclusions:**

IVGC, alone or combination with OR, and teprotumumab should be preferred as the most effective strategies for active moderate to severe TED. Teprotumumab showed profound effect on proptosis reduction. OIGC, OR, and somatostatin analogs showed some statistical benefit and can be employed as second-line treatment strategies. RTX is a promising biologic agent, but more RCTs are required to define its appropriate role in treating TED.

## 1. Introduction

Thyroid eye disease (TED), also called Graves' ophthalmopathy, is the most common autoimmune orbital disease in adults. Epidemiological studies have revealed that the majority of TED patients have stable and mild eye sign, but approximately 3%–6% of TED patients progress to an active moderate-to-severe phase with intense orbital pain, inflammation, motility restriction, and even sight-threatening corneal ulceration or compressive optic neuropathy [1, 2].

The pathogenesis of TED is complex and remains unclear. This has limited the development of therapies targeting the underlying cause of the disease. Traditional treatment strategies include corticosteroids via different administration routes (intravenous, oral, or orbital injection), orbital radiotherapy (OR), and a combination of glucocorticoids with OR. In recent years, some biologic agents such as teprotumumab, rituximab, tocilizumab, and etanercept have been introduced to treat active, moderate to severe TED [3, 4], and some antioxidants like selenium have shown to significantly improve the quality of life and reduce the risk of disease progression [5]. A number of randomized controlled trials (RCTs) have been conducted to compare the efficacy of these treatment strategies; however, there has been no consensus as to the best treatment strategy.

Several traditional meta-analyses examining different treatment strategies for TED have been published [6–9], but each has some critical limitations. Since conventional meta-analysis can only compare two strategies, it is difficult to integrate all available data to generate a clear hierarchy among all treatment modalities. Moreover, it is impossible to compare more than two strategies simultaneously. For these reasons, there is a need to perform a network meta-analysis which allows the integration of data from direct and indirect comparisons to provide evidence-based suggestions for treatment decision-making. Herein, we did a network meta-analysis using the frequent method to comprehensively compare and rank treatments for TED based on published RCT data.

## 2. Materials and Methods

### 2.1. Study Eligibility and Identification

This network meta-analysis protocol was registered at PROSPERO (number CRD42017058612). PubMed, Embase, Web of Science, Google Scholar, and CENTRAL were searched (up to May 2017) to identify relevant RCTs. In addition, we also searched the reference lists of published meta-analyses and reviewed articles manually. All articles comparing any treatment strategy with a placebo or another strategy for the treatment of active TED were recruited. The trials enrolled in the network meta-analysis meet the following criteria: the study design was RCT; participants had active TED; efficacy outcomes included response rate, proptosis, and disease activity. Trials were excluded as follows: treatment strategies for patients with hyperthyroidism; surgical treatment for TED; study outcomes not containing response rate; duplicated publications.

### 2.2. Outcomes Measures, Data Extraction, and Quality Assessment

The primary outcome was the response rate which was defined as clinical success or improvement as assessed by each trial. Secondary outcome was the reduction in proptosis from the baseline to the end of follow-up. Another outcome was the reduction in the disease activity from the baseline to the end of follow-up. Safety outcomes were accessed by calculating the proportion of all adverse events.

Two investigators (NX and YC) selected studies and extracted the data from each included trial independently. The following information was obtained: authors, publication date, study design, intervention and dose, sample size, disease stage, mean age, sex ratio, and follow-up. Any discrepancies about inclusion between the two reviewers were discussed with a third reviewer (TLX or MZ) until a consensus was reached.

The Cochrane risk of bias assessment tool was used to assess the risk of bias of each study. The risk of bias includes seven components: random sequence generation, allocation concealment, blinding of participants and personnel, blinding of outcome assessment, incomplete outcome data, selective reporting, and other bias.

### 2.3. Statistical Analyses

In order to make comparisons between each treatment strategy, a random-effects network model using the frequent method was built. All data calculations were performed using the STATA13.1. A network plot was employed to represent the overall information of the trials included in the analysis. Nodes size represented the number of trials for each strategy, and line thickness represented the number of direct comparisons. Inconsistencies were tested by calculating the inconsistency factor (IF) and ratio of two odds ratios (RoR) among studies in each closed loop. When the 95% CIs of an RoR value reached one, no statistical inconsistency was considered to exist for this trial. A forest plot containing confidence intervals (CIs) and corresponding predictive intervals (PrI) was used to summarize the relative mean effects and predictions of each comparison. Traditional funnel plots were drawn to confirm the risk of publication bias for all included trials. Network ranking was used to measure the probability of the best treatment choice among all strategies. Treatment strategies with a greater value in the histogram were associated with a greater probability of superior efficacy.

## 3. Results

### 3.1. Literature Search, Characteristics, and Quality Assessment

**Figure 1:**
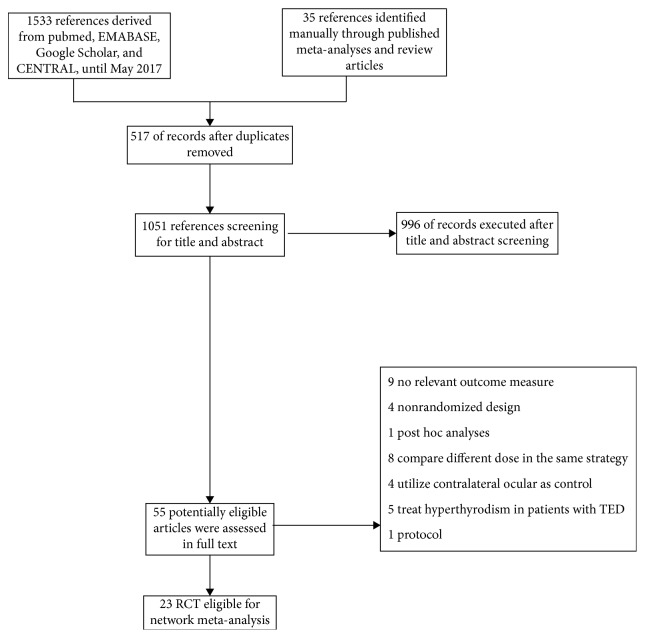
Flowchart of RCT searches.

As shown in Figure 1, a total of 1568 citations were identified, and the full texts of 55 potentially eligible articles were assessed after title and abstract screening. Subsequently, 32 studies were excluded after complete reading of the articles.  Table 1 presents the characteristics of included trials. Overall, twenty-three trials with a total sample size of 1047 patients published between 1983 and 2017 were recruited for this network meta-analysis. We merged sham radiation and placebo groups from each study into a placebo group, thus a total of eleven different medical treatment strategies and placebo were identified, including intravenous glucocorticoids (IVGC) [10–15], oral glucocorticoids (OGC) [10, 12, 14–21], orbital injection of glucocorticoids (OIGC) [16], OR [22, 23], IVGC combined with OR (IVGC + OR) [13, 24], OGC combined with OR (OGC + OR) [21, 24, 25], rituximab (RTX) [26, 27], somatostatin analogs [28–31], intravenous immunoglobulin (IVIG) [18], teprotumumab [4], and cyclosporine [20].

**Figure 2:**
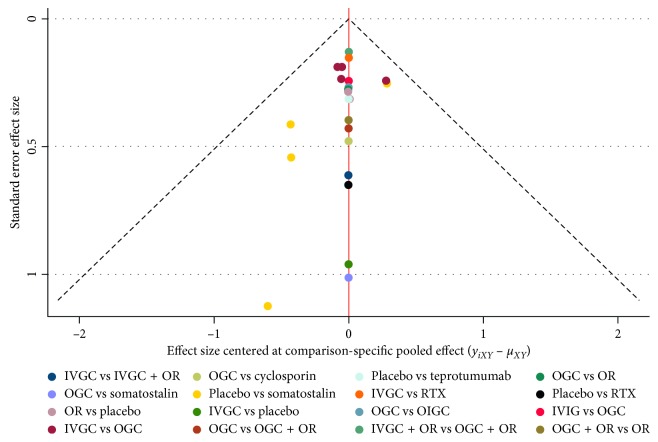
Funnel plot for the network meta-analysis.

The biases of the 23 included studies were assessed using the Cochrane risk of bias assessment tool and a funnel plot. As shown in Supplementary Figure 1, 12 studies (54.5%) described random sequence generation and blinding of participants and personnel, 11 studies (50%) had allocation concealment, 17 studies (77.3%) had a low risk of blinding of the outcome assessment and selective reporting, and 20 studies (90.1%) had a low risk of incomplete outcome data. Most studies (75%) were regarded as having unclear risks of bias in the domain of other biases.  Figure 2 shows that all included studies are symmetrically distributed around the vertical line (*x* = 0) in a funnel plot, implying there was no significant publications bias.

### 3.2. Evidence Network and Inconsistency Plots

**Figure 3:**
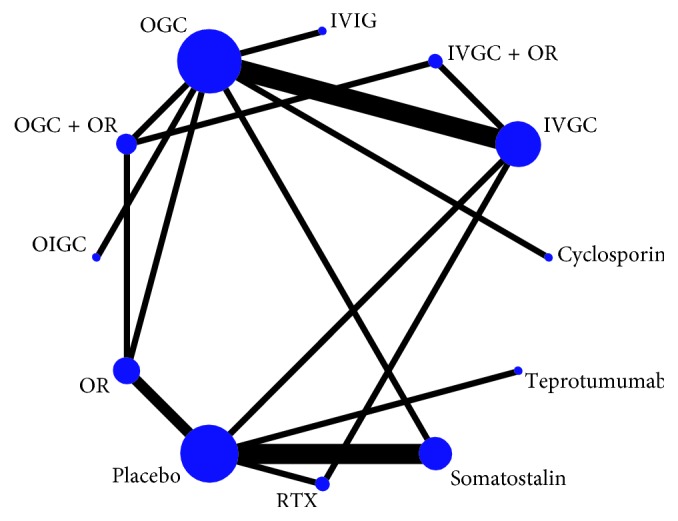
The evidence network of all enrolled RCTs about ten strategies in this network meta-analysis. Nodes size represents the number of trials for each strategy and lines thickness represents the number of direct comparisons.

The evidence network of eligible comparisons is shown in Figure 3. The most studied treatment strategies were IVGC, OGC, and placebo. As shown in Supplementary Figure 2, the inconsistency plot consists of two triangular loops and four quadrangular loops. The 95% CI of RoR values of all loops were truncated at zero, and *P* value > 0.05 verified their consistency statistically. However, the mean RoR value was 8.029, and *P*=0.075 for the IVGC-Placebo-RTX loop, indicating some heterogeneity in this loop.

## 4. Efficacy

### 4.1. Response Rate

**Figure 4:**
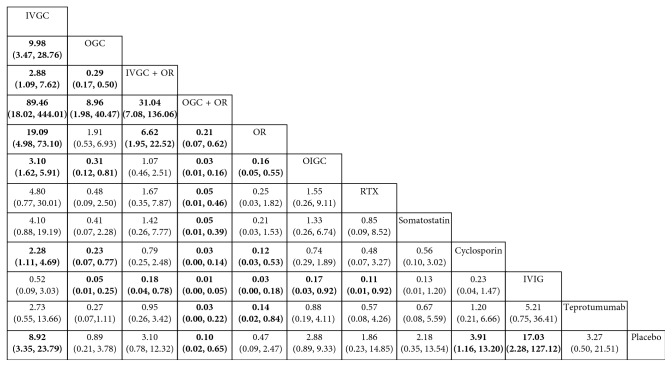
The confidence intervals of estimates for the network analysis. The bold and underlined data indicate that there are statistically significant effects. (*P* < 0.05).

**Figure 5:**
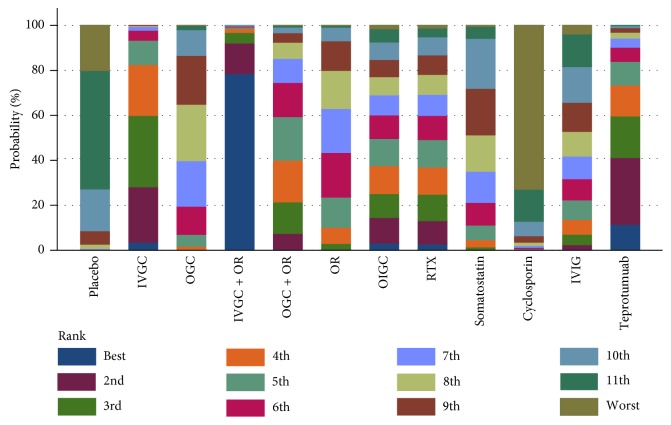
Rank probability of each treatment strategies for response rate in the network analysis.

Figure 4 summarizes the response rate with 95% CI for all treatment strategies that combine direct and indirect comparisons. The ranking graphs of probability distribution of response rates are shown in Figure 5. Rank probability analysis showed that IVGC + OR ranked highest, followed by teprotumumab, IVGC, OGC + OR, RTX, OIGC, OR, IVIG, OGC, somatostatin, placebo, and cyclosporine.

### 4.2. Proptosis Reduction

**Figure 6:**
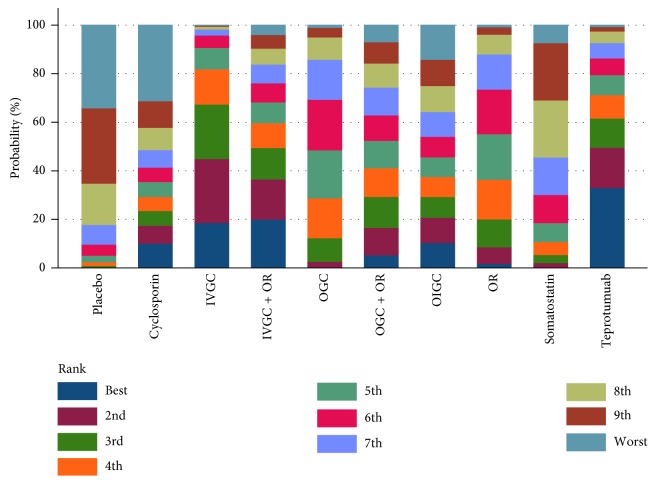
Rank probability of each treatment strategies for proptosis reduction in the network analysis.

Sixteen studies mentioned proptosis reduction from baseline to the end of the follow-up. Studies referring to RTX and IVIG are not included; thus, we were not able to rank these two strategies in the dimension of proptosis. Rank probability analysis showed that teprotumumab ranked highest, followed by IVGC, IVGC + OR, OIGC, OGC, OGC + OR, OR, somatostatin, cyclosporine, and placebo (Figure 6).

### 4.3. Reduction in Disease Activity

Nineteen studies mentioned a reduction in the disease activity from baseline to the end of follow-up. There were five kinds of measurements of activity. Six studies used the 10-point clinical activity scores (CAS), eight studies used the 7-point CAS, one study used the 8-point CAS, two studies used the total eye scores (TES), and two studies used the ophthalmopathy index (OI).  Table 2 presented direct comparisons using the same measurement of activity. IVGC was demonstrated to be significantly better than OGC and placebo, while teprotumumab was significantly better than placebo. IVGC + OR was significantly better than OGC + OR. OGC + OR was significantly better than OGC. There was no statistically significant difference between the other groups.

### 4.4. Overall Adverse Events

Table 3 summarized the crude rates of adverse events mentioned in conjunction with the eleven treatment strategies analyzed. The crude rate of adverse events was relatively high in the OGC group. IVGC and cyclosporine were demonstrated to induce serious adverse effects like liver dysfunction. Teprotumumab was associated with a high rate in minor events. OR and OIGC were reported to have local and mild complications. RTX was shown to be relatively safe with some complications of infusion reactions. Additionally, somatostatin had a high rate in gastrointestinal complications. No serious adverse effects were reported with IVIG.

## 5. Discussion

This network meta-analysis represents a comprehensive synthesis of data for eleven different medical treatment strategies and placebo for active TED. The results of our analysis showed that IVGC + OR had the highest probability to be the best treatment in terms of response rate, follow by teprotumumab and IVGC. In terms of proptosis reduction, teprotumumab and IVGC were identified as the best strategy. These findings were surprising because it is different to previous studies. Teprotumumab is a fully human IGF-IR inhibitory monoclonal antibody that received a “breakthrough therapy” designation from the Food and Drug Administration in 2016. The most striking result was that patients who received teprotumumab had reductions in proptosis that were more effective than other strategies. In this trial, proptosis reduced by an average of 2.46 mm (vs. 0.15 mm in placebo). In contrast, proptosis average decrease was less than 1 mm in IVGC, even using the highest dose [32]. However, teprotumumab was not enclosed in a comparative loop in this analysis, which may lessen the statistical power. Further head to head trial comparing teprotumumab and gold standard treatment like IVGC may enhance the value of this novel strategy. Furthermore, the side effect profile of the treatment remains to be determined in larger studies, e.g., on the interaction with glycemic control.

Recent published meta-analysis comparing common immunosuppressive therapies for TED concluded that IVGC is an effective treatment and has a significant advantage over OGC with fewer adverse events [6, 8]. Stiebel-Kalish et al. found that a combination of glucocorticoids and OR was better than either treatment alone in a meta-analysis comparing all treatment modalities for TED [7]. In 2016, the European Group on Graves' ophthalmopathy (EUGOGO) suggested that high-dose IVGC should be considered as first-line treatment for moderate to severe and active GO [33]. The effectiveness of IVGC, alone or combined with OR, may be due to the higher single and cumulative treatment dosage which may better suppress immune function and decrease inflammation [34]. A RCT performed by EUGOGO compared three different cumulative doses (2.25, 4.98, and 7.47 g) of IVGC and found that 7.47 g provided short-term advantages over lower doses, but this benefit did not persist at 24 weeks and was associated with adverse events [32].

OIGC ranked behind the systemic use of glucocorticoids. Administration of triamcinolone by orbital injection allows its diffusion into the extraocular muscles, leading to a reduction in diplopia and inflammatory signs [35, 36]. OIGC can improve the symptoms of inflammation without the unacceptable rates of local complications, but this treatment has not been proven to be as effective as systemic glucocorticoids therapy in our study. It is necessary to mention that comparing OIGC directly with systemic glucocorticoids undermines the specific efficacy of this treatment which may be indicated following IVGC patients with unbearable side effects or in recurrences. On the contrary, OIGC was not enclosed in a comparative loop in this analysis. Although there were two RCTs examining OIGC, the one comparing OIGC and placebo was not enrolled in our network meta-analysis due to irrelevant outcome measurements [36]. Thus, the ranking of this strategy should be identified as uncertain.

Except for the use of glucocorticoids, evidence for efficacy of other treatment strategies remain unclear. OR showed minor but statistically significant advantages over placebo, and its efficacy ranked in the middle of all strategies. Although OR has been used to treat TED for over 60 years and the rate of severe side effect are rare [37], conflicting results from several studies have called its efficacy into question [38]. A recent survey sponsored by American Society of Ophthalmic Plastic and Reconstructive Surgery (ASOPRS) showed that while 70% of doctors used OR, only 2% used it as first-line treatment, 20% as second-line treatment, and 33% as third-line treatment [39]. Steibel-Kalish et al. and Tanda et al. summarized that OR may have a modest effect on extraocular muscle motility in early TED disease process, and its effectiveness can be increased by the synergistic interaction with glucocorticoids [7, 40]. Our results indicating that IVGC + OR ranked the best in terms of response rate supported these findings. Similarly, the efficacy of somatostatin analogs was superior to placebo statistically but without clinical significance. Given its gastrointestinal side effects and high cost, its use was not recommended as first-line treatment by EUGOGO [33]. On the contrary, cyclosporine and IVIG did not show any statistical efficacy when compared with placebo. Thus, they should also not be recommended for routine use for TED.

The results of inconsistency testing showed that the 95% CI of IVGC-Placebo-RTX loop RoR value is truncated at one, but the average RoR value is 8.029. Large RoR values signify significant inconsistency in this loop. RTX, a monoclonal antibody directed against CD20 on B lymphocytes, is the key node in this loop and has showed some encouraging effect on active moderate to severe TED as well as some IVGC-resistant cases [41, 42]. However, the two published RCTs of RTX had discrepant outcomes. RTX was shown to offer no additional benefit over placebo in the first trial by Stan et al. but was slightly better than IVGC in the second study by Salvi et al. Since it has been generally proven and accepted that IVGC is more efficacious than placebo, the differences of these two trials need to be carefully discussed. We observed that there were obvious differences in baseline data between each of these trials. First, the disease duration in the study by Stan's et al. (mean 373 days, vs. 299 days) is longer than Salvi et al.'s study (mean 45 months vs. 46 months). According to Rundle's curve [43], active TED patients might improve spontaneously to inactive, fibrotic stage with time elapse. Second, previous research has shown that age and gender may influence the disease course in TED [44]. It should be noted that the mean age is older and female ratio is less in Stan et al. vs. Salvi et al., which means there is a lower likelihood of response to therapy in the Stan study. Finally, other factors such as the interval of previous corticosteroids use, the smoking proportion of included patients, and TRAb levels in each study were different. It remains unclear to what extent did these differences affect the results of comparison [45].

There were some limitations in this network meta-analysis that should be noted. It is generally accepted that selection of the appropriate treatment strategy for TED depends on an important outcome measurement: the reduction of clinical activity score (CAS). However, the eligible studies utilized different methods to calculate the activity of TED, including the 10-point CAS [10–12, 22, 23, 30, 31], 7-point modified CAS [4, 14, 17, 24, 26–29], 8-point CAS [16], total eye scores (TES) [13, 19, 20], and ophthalmopathy index (OI) [15, 21, 25]. This high diversity of disease activity assessment makes it difficult to combine the data and conduct accurate ranking for treatment strategies. Proptosis reduction outcomes were not reported in some studies such as RTX and IVIG, so that we were not able to assess the ranking of these two strategies in terms of this outcome. In addition, the sample size of eligible trials was relatively small, especially for the combination of corticosteroids and OR, which prevented stronger conclusions. Furthermore, in order to merge the outcomes in a unified format, some well-designed RCTs exploring other promising treatment strategies such as selenium [4], plasma filtration [46], colchicines [47], and OGC combined with cyclosporine [48] were not included in this analysis.

Based on the results of this network meta-analysis of eleven medical treatment strategies for TED, several conclusions can be drawn: IVGC, alone or combination with OR, and teprotumumab should be preferred as the most effective strategies for active moderate to severe TED. teprotumumab showed profound effects on proptosis reduction. OIGC, OR, and somatostatin analogs showed some statistical benefit and may serve as a second-line treatment strategy. RTX is a promising biologic agent, but more RCTs are required to better define its appropriate role in treating TED.

## Figures and Tables

**Table 1:** Study characteristics of included RCTs.

Authors	Year	Country	Design	Intervention	Sample size	Stage	Mean age	Sex (M/F)	Follow-up (month)
Macchia et al. [15]	2001	Italy	OL-P	IVGC vs. OGC	25 vs. 26	OI 4.43 vs. 2.65	42.6 vs. 44.57	11/40	12
Kahaly et al. [14]	2005	Germany	SB-P	IVGC vs. OGC	35 vs. 35	CAS (7) 5 vs. 5	52 vs. 48	21/49	6
Aktaran et al. [12]	2007	Turkey	SB-P	IVGC vs. OGC	25 vs. 27	CAS (10) 5.2 vs. 5.0	44.3 vs. 41.3	24/28	3
Roy et al. [10]	2015	Indian	OL-P	IVGC vs. OGC	31 vs. 31	CAS (10) 4.29 vs. 3.94	37.61 vs. 36.93	24/38	12
van Geest et al. [11]	2008	Netherlands	DB-P	IVGC vs. placebo	6 vs. 9	CAS (10) 6 vs. 4.9	50.7 vs. 44.7	3/12	12–48
Salvi et al. [27]	2015	Italy	DB-P	Rtx vs. IVGC	15 vs. 16	CAS (7) 4.4 vs. 4.7	51.9 vs. 50.4	5/26	6–12
Stan et al. [26]	2015	America	DB-P	Rtx vs. placebo	12 vs. 13	CAS (7) 4.9 vs. 5.3	57.6 vs. 61.8	8/17	6–12
Ng et al. [13]	2005	Hongkong	SB-P	IVGC vs. IVGC + OR	8 vs. 8	TES 16.5 vs. 18	48.3 vs. 64.1	10/6	12
Marcocci et al. [24]	2001	Italy	SB-P	IVGC + OR vs. OGC + OR	41 vs. 41	CAS (7) 4.5 vs. 4.2	50 vs. 48	14/68	12
Alkawas et al. [16]	2010	Egypt	OL-P	OGC vs. OIGC	15 vs. 14	CAS (8) 4.75 vs. 5	N/A	8/16	6
Marcocci et al. [25]	1991	Italy	OL-P	OGC + OR vs. OR	13 vs. 13	OI 5.85 vs. 5.46	47.3 vs. 46	8/18	6–18
Prummel et al. [19]	1993	Netherlands	DB-P	OGC vs. OR	28 vs. 28	TES 8.7 vs. 9.4	47 vs. 46.6	9/47	6
Bartalena et al. [21]	1983	Italy	SB-P	OGC + OR vs. OGC	12 vs. 12	OI 6.4 vs. 6.2	42 vs. 46	11/13	12–28
Mourits et al. [23]	2000	Netherlands	DB-P	OR vs. placebo	30 vs. 30	CAS (10) 3.3 vs. 3.4	48.7 vs. 49	9/51	6
Prummel et al. [22]	2004	Netherlands	DB-P	OR vs. placebo	44 vs. 44	CAS (10) 3.0 vs. 3.3	45.2 vs. 45.1	18/70	12
Stan et al. [28]	2006	America	DB-P	SSAnalogs vs. placebo	14 vs. 11	CAS (7) 6 vs. 5	53 vs. 61	7/18	4
Chang and Liao [29]	2006	Taiwan	DB-P	SSAnalogs vs. placebo	30 vs. 30	CAS (7) 3.6 vs. 3.7	43.0 vs. 43.1	17/43	3
Dickinson et al. [31]	2004	UK	DB-P	SSAnalogs vs. placebo	23 vs. 27	CAS (10) 5.39 vs. 5.85	50 vs. 50	11/39	4
Wémeau et al. [30]	2005	France	DB-P	SSAnalogs vs. placebo	25 vs. 25	CAS (10) 4.2 vs. 4.5	47.5 vs. 47.1	10/40	6
Kung et al. [17]	1996	Hongkong	OL-P	SSAnalogs vs. OGC	8 vs. 10	CAS (7) 5 vs. 3	38.2 vs. 45.2	9/9	3
Prummel et al. [20]	1989	Netherlands	SB-P	OGC vs. cyclosporin	18 vs. 18	TES 12.9 vs. 11.5	49 vs. 52	10/26	3
Kahaly et al. [18]	1996	Germany	OL-P	OGC vs. IVIG	19 vs. 21	N/A	47 vs. 48	9/31	5
Smith et al. [4]	2017	America	DB-P	Teprotumumab vs. placebo	42 vs. 45	CAS (7) 5.1 vs. 5.2	51.6 vs. 54.2	83/64	6

**Table 2:** Outcomes of direct comparisons in disease activity.

Outcome of disease activity	Comparisons	No. of comparisons	CAS reduction (MD, 95% CI)
7-point CAS	IVGC vs. OGC	1	1.00 [−1.10, 3.10]
	Rtx vs. IVGC	1	1.40 [−0.01, 2.81]
	Rtx vs. placebo	1	0.1 [−1.47, 1.67]
	IVGC + OR vs. OGC + OR	1	**0.80 [0.31, 1.29]**
	Somastatin vs. placebo	2	0.72 [−0.09, 1.52]
	Somastatin vs. OGC	1	1.00 [0.06, 1.94]
	Teprotumumab vs. placebo	1	**1.58 [1.51, 1.65]**

10-point CAS	IVGC vs. OGC	2	**1.13 [0.40, 1.86]**
	IVGC vs. placebo	1	**3.49 [2.25, 4.73]**
	OR vs. placebo	1	−0.35 [−0.92, 0.22]
	SS vs. placebo	2	0.25 [−0.53, 1.04]

8-point CAS	OGC vs. OIGC	1	−0.25 [−1.12, 0.62]

TES	IVGC vs. IVGC + OR	1	−4.00 [−11.59, 3.59]
	OGC vs. cyclosporin	1	3.60 [−0.45, 7.65]

OI	IVGC vs. OGC	1	**1.11 [0.30, 1.92]**
	OGC + OR vs. OGC	1	**2.51 [1.40, 3.63]**

Significant results are in bold.

**Table 3:** Overall adverse events in all treatment strategies.

Adverse events	Constituent ratio										
	IVGC	OGC	OIGC	OR	IVGC+OR	OGC + OR	Rtx	Somatostatin	IVIG	Cyclosporine	Teprotumumab
Major event											
Liver dysfunction	7/88	0/66	N/A	N/A	1/41	N/A	N/A	N/A	0/21	1/18	N/A
Cushingoid features	7/97	27/156	N/A	0/28	9/49	35/41	N/A	N/A	0/21	0/18	N/A
Weight gain	13/122	39/171	0/14	3/28	4/8	N/A	N/A	N/A	0/21	3/18	N/A
Gastrointestinal	22/138	11/112	1/14	2/28	7/49	4/41	2/13	60/82	0/21	N/A	9/43
Hypertension	5/97	23/181	1/14	0/28	4/49	2/41	N/A	N/A	0/21	6/18	N/A
Hyperglycaemia	11/113	7/145	0/14	N/A	9/41	11/49	N/A	N/A	0/21	0/18	5/43
Inflammatory bowel disease	N/A	N/A	N/A	N/A	N/A	N/A	N/A	N/A	N/A	N/A	1/43
*Escherichia* sepsis	N/A	N/A	N/A	N/A	N/A	N/A	N/A	N/A	N/A	N/A	1/43
Urinary retention	N/A	N/A	N/A	N/A	N/A	N/A	N/A	N/A	N/A	N/A	1/43
Minor event	32/215	23/276	N/A	30/170	21/110	10/41	7/54	N/A	0/21	0/18	32/74

## Supplementary Materials

Supplementary Materials The supplementary materials include two figures: Figure 1: risk of bias of the included RCTs. Figure 2: inconsistency test for direct and indirect comparison in the network meta-analysis.

## References

[1] Tanda M. L., Piantanida E., Liparulo L. (2013). Prevalence and natural history of graves’ orbitopathy in a large series of patients with newly diagnosed graves’ hyperthyroidism seen at a single center. *Journal of Clinical Endocrinology & Metabolism*.

[2] Wiersinga W. M., Bartalena L. (2002). Epidemiology and prevention of graves’ ophthalmopathy. *Thyroid*.

[3] Wiersinga W. M. (2017). Advances in treatment of active, moderate-to-severe graves’ ophthalmopathy. *Lancet Diabetes & Endocrinology*.

[4] Smith T. J., Kahaly G. J., Ezra D. G. (2017). Teprotumumab for thyroid-associated ophthalmopathy. *New England Journal of Medicine*.

[5] Marcocci C., Kahaly G. J., Krassas G. E. (2011). Selenium and the course of mild graves’ orbitopathy. *New England Journal of Medicine*.

[6] Mou P., Jiang L. H., Zhang Y. (2015). Common immunosuppressive monotherapy for graves’ ophthalmopathy: a meta-analysis. *PLoS One*.

[7] Stiebel-Kalish H., Robenshtok E., Hasanreisoglu M., Ezrachi D., Shimon I., Leibovici L. (2009). Treatment modalities for graves’ ophthalmopathy: systematic review and metaanalysis. *Journal of Clinical Endocrinology & Metabolism*.

[8] Gao G., Dai J., Qian Y., Ma F. (2014). Meta-analysis of methylprednisolone pulse therapy for graves’ ophthalmopathy. *Clinical & Experimental Ophthalmology*.

[9] Viani G. A., Boin A. C., De Fendi L. I., Fonseca E. C., Stefano E. J., Paula J. S. d. (2012). Radiation therapy for graves’ ophthalmopathy: a systematic review and meta-analysis of randomized controlled trials. *Arquivos Brasileiros de Oftalmologia*.

[10] Roy A., Dutta D., Ghosh S., Mukhopadhyay P., Mukhopadhyay S., Chowdhury S. (2015). Efficacy and safety of low dose oral prednisolone as compared to pulse intravenous methylprednisolone in managing moderate severe graves’ orbitopathy: a randomized controlled trial. *Indian Journal of Endocrinology and Metabolism*.

[11] van Geest R. J., Sasim I. V., Koppeschaar H. P. F. (2008). Methylprednisolone pulse therapy for patients with moderately severe graves’ orbitopathy: a prospective, randomized, placebo-controlled study. *European Journal of Endocrinology*.

[12] Aktaran S., Akarsu E., Erbağci I., Araz M., Okumuş S., Kartal M. (2007). Comparison of intravenous methylprednisolone therapy vs. oral methylprednisolone therapy in patients with graves’ ophthalmopathy. *International Journal of Clinical Practice*.

[13] Ng C. M., Yuen H. K., Choi K. L. (2005). Combined orbital irradiation and systemic steroids compared with systemic steroids alone in the management of moderate-to-severe graves’ ophthalmopathy: a preliminary study. *Hong Kong Medical Journal*.

[14] Kahaly G. J., Pitz S., Hommel G., Dittmar M. (2005). Randomized, single blind trial of IntravenousversusOral steroid monotherapy in graves’ orbitopathy. *Journal of Clinical Endocrinology & Metabolism*.

[15] Macchia P. E., Bagattini M., Lupoli G., Vitale M., Vitale G., Fenzi G. (2001). High-dose intravenous corticosteroid therapy for graves’ ophthalmopathy. *Journal of Endocrinological Investigation*.

[16] Alkawas A. A., Hussein A. M., Shahien E. A. (2010). Orbital steroid injection versus oral steroid therapy in management of thyroid-related ophthalmopathy. *Clinical & Experimental Ophthalmology*.

[17] Kung A. W. C., Michon J., Tai K. S., Chan F. L. (1996). The effect of somatostatin versus corticosteroid in the treatment of graves’ ophthalmopathy. *Thyroid*.

[18] Kahaly G., Pitz S., Muller-forell W., Hommel G. (1996). Randomized trial of intravenous immunoglobulins versus prednisolone in graves’ ophthalmopathy. *Clinical and Experimental Immunology*.

[19] Prummel M. F., Berghout A., Wiersinga W. M., Mourits M. P., Koornneef L., Blank L. (1993). Randomised double-blind trial of prednisone versus radiotherapy in graves’ ophthalmopathy. *The Lancet*.

[20] Prummel M. F., Mourits M. P., Berghout A. (1989). Prednisone and cyclosporine in the treatment of severe graves’ ophthalmopathy. *New England Journal of Medicine*.

[21] Bartalena L., Marcocci C., Chiovato L. (1983). Orbital cobalt irradiation combined with systemic corticosteroids for graves’ ophthalmopathy: comparison with systemic corticosteroids alone^*∗*^. *Journal of Clinical Endocrinology & Metabolism*.

[22] Prummel M. F., Terwee C. B., Gerding M. N. (2004). A randomized controlled trial of orbital RadiotherapyVersusSham irradiation in patients with mild graves’ ophthalmopathy. *Journal of Clinical Endocrinology & Metabolism*.

[23] Mourits M. P., van Kempen-Harteveld M. L., García M. B. G., Koppeschaar H. P., Tick L., Terwee C. B. (2000). Radiotherapy for graves’ orbitopathy: randomised placebo-controlled study. *The Lancet*.

[24] Marcocci C., Bartalena L., Tanda M. L (2001). Comparison of the effectiveness and tolerability of intravenous or oral glucocorticoids associated with orbital radiotherapy in the management of severe graves’ ophthalmopathy: results of a prospective, single-blind, randomized study. *Journal of Clinical Endocrinology & Metabolism*.

[25] Marcocci C., Bartalena L., Bogazzi F., Bruno-Bossio G., Lepri A., Pinchera A. (1991). Orbital radiotherapy combined with high dose systemic glucocorticoids for graves’ ophthalmopathy is more effective than radiotherapy alone: results of a prospective randomized study. *Journal of Endocrinological Investigation*.

[26] Stan M. N., Garrity J. A., Carranza Leon B. G., Prabin T., Bradley E. A., Bahn R. S. (2015). Randomized controlled trial of rituximab in patients with graves’ orbitopathy. *Journal of Clinical Endocrinology & Metabolism*.

[27] Salvi M., Vannucchi G., Currò N. (2015). Efficacy of B-cell targeted therapy with rituximab in patients with active moderate to severe graves’ orbitopathy: a randomized controlled study. *The Journal of Clinical Endocrinology & Metabolism*.

[28] Stan M. N., Garrity J. A., Bradley E. A. (2006). Randomized, double-blind, placebo-controlled trial of long-acting release octreotide for treatment of graves’ ophthalmopathy. *Journal of Clinical Endocrinology & Metabolism*.

[29] Chang T.-C., Liao S.-L. (2006). Slow-release lanreotide in graves’ ophthalmopathy: a double-blind randomized, placebo-controlled clinical trial. *Journal of Endocrinological Investigation*.

[30] Wémeau J. L., Caron P., Beckers A. (2005). Octreotide (long-acting release formulation) treatment in patients with graves’ orbitopathy: clinical results of a four-month, randomized, placebo-controlled, double-blind study. *The Journal of Clinical Endocrinology & Metabolism*.

[31] Dickinson A. J., Vaidya B., Miller M. (2004). Double-blind, placebo-controlled trial of octreotide long-acting repeatable (LAR) in thyroid-associated ophthalmopathy. *Journal of Clinical Endocrinology & Metabolism*.

[32] Bartalena L., Krassas G. E., Wiersinga W. (2012). Efficacy and safety of three different cumulative doses of intravenous methylprednisolone for moderate to severe and active graves’ orbitopathy. *The Journal of Clinical Endocrinology & Metabolism*.

[33] Bartalena L., Baldeschi L., Boboridis K. (2016). The 2016 european thyroid association/european group on graves’ orbitopathy guidelines for the management of graves’ orbitopathy. *European Thyroid Journal*.

[34] Zang S., Ponto K. A., Kahaly G. J. (2011). Intravenous glucocorticoids for graves’ orbitopathy: efficacy and morbidity. *The Journal of Clinical Endocrinology & Metabolism*.

[35] Bordaberry M., Marques D. L., Pereira-Lima J. C., Marcon I. M., Schmid H. (2009). Repeated peribulbar injections of triamcinolone acetonide: a successful and safe treatment for moderate to severe graves’ ophthalmopathy. *Acta Ophthalmologica*.

[36] Ebner R., Devoto M. H., Weil D. (2004). Treatment of thyroid associated ophthalmopathy with periocular injections of triamcinolone. *British Journal of Ophthalmology*.

[37] Schaefer U., Hesselmann S., Micke O. (2002). A long-term follow-up study after retro-orbital irradiation for graves’ ophthalmopathy. *International Journal of Radiation Oncology∗Biology∗Physics*.

[38] Bradley E. A., Gower E. W., Bradley D. J. (2008). Orbital radiation for graves ophthalmopathy. *Ophthalmology*.

[39] Perumal B., Meyer D. R. (2015). Treatment of severe thyroid eye disease. *Ophthalmic Plastic and Reconstructive Surgery*.

[40] Tanda M. L., Bartalena L. (2012). Efficacy and safety of orbital radiotherapy for graves’ orbitopathy. *Journal of Clinical Endocrinology & Metabolism*.

[41] Salvi M., Vannucchi G., Beck-Peccoz P. (2013). Potential utility of rituximab for graves’ orbitopathy. *The Journal of Clinical Endocrinology & Metabolism*.

[42] Salvi M., Vannucchi G., Campi I. (2007). Treatment of graves’ disease and associated ophthalmopathy with the anti-CD20 monoclonal antibody rituximab: an open study. *European Journal of Endocrinology*.

[43] Rundle F. F. (1957). Management of exophthalmos and related ocular changes in graves’ disease. *Metabolism: Clinical and Experimental*.

[44] Perros P., Crombie A. L., Matthews J. N. S., Kendall-Taylor P. (1993). Age and gender influence the severity of thyroid-associated ophthalmopathy: a study of 101 patients attending a combined thyroid-eye clinic. *Clinical Endocrinology*.

[45] Stan M. N., Salvi M. (2017). Management of endocrine disease: rituximab therapy for graves’ orbitopathy-lessons from randomized control trials. *European Journal of Endocrinology*.

[46] Cap J., Ceeova V., Skacha M., Rezek P., Vlcek P., Blaha M. (2010). Plasma filtration in the treatment of graves’ ophthalmopathy: a randomized study. *Journal of Clinical Apheresis*.

[47] Stamato F. J. D. C., Maciel R. M. D. B., Manso P. G. (2006). Colchicine in the treatment of the inflammatory phase of graves’ ophthalmopathy: a prospective and randomized trial with prednisone. *Arquivos Brasileiros de Oftalmologia*.

[48] Kahaly G., Schrezenmeir J., Krause U. (1986). Ciclosporin and prednisone v. prednisone in treatment of graves’ ophthalmopathy: a controlled, randomized and prospective study. *European Journal of Clinical Investigation*.

