# Supplementary figures and images for: Comparative Efficacy of Medical Treatments for Thyroid Eye Disease: A Network Meta-Analysis
Source: J Ophthalmol. 2018 Dec 12;2018:7184163. doi: 10.1155/2018/7184163 (PMC6311851; doi:10.1155/2018/7184163)

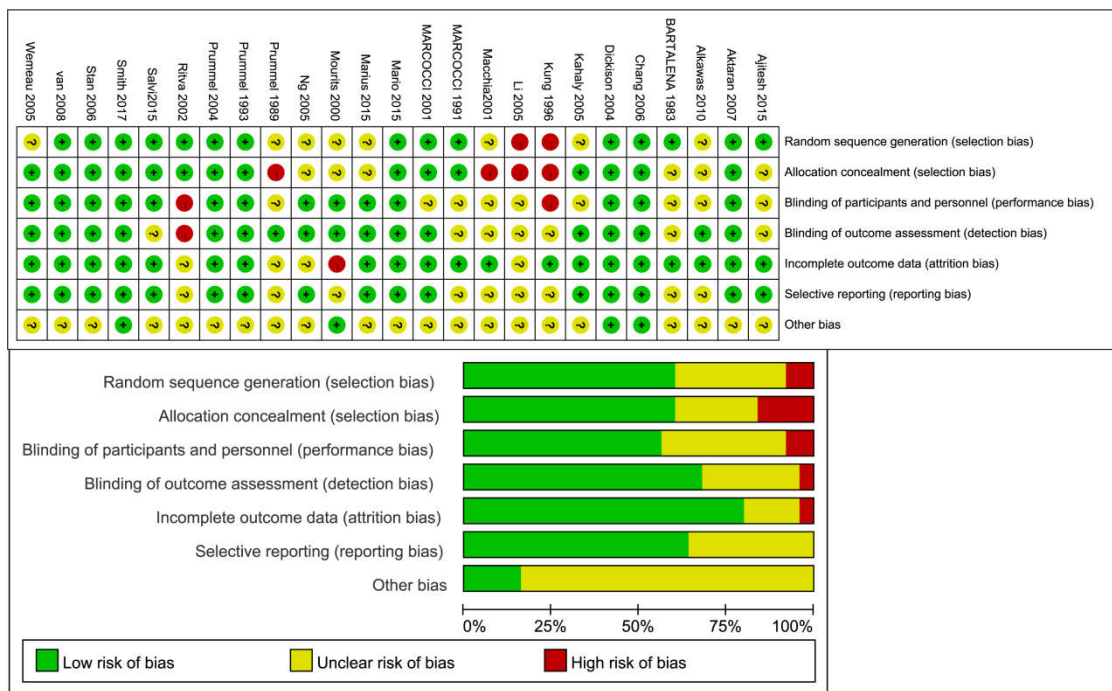

Fig.1

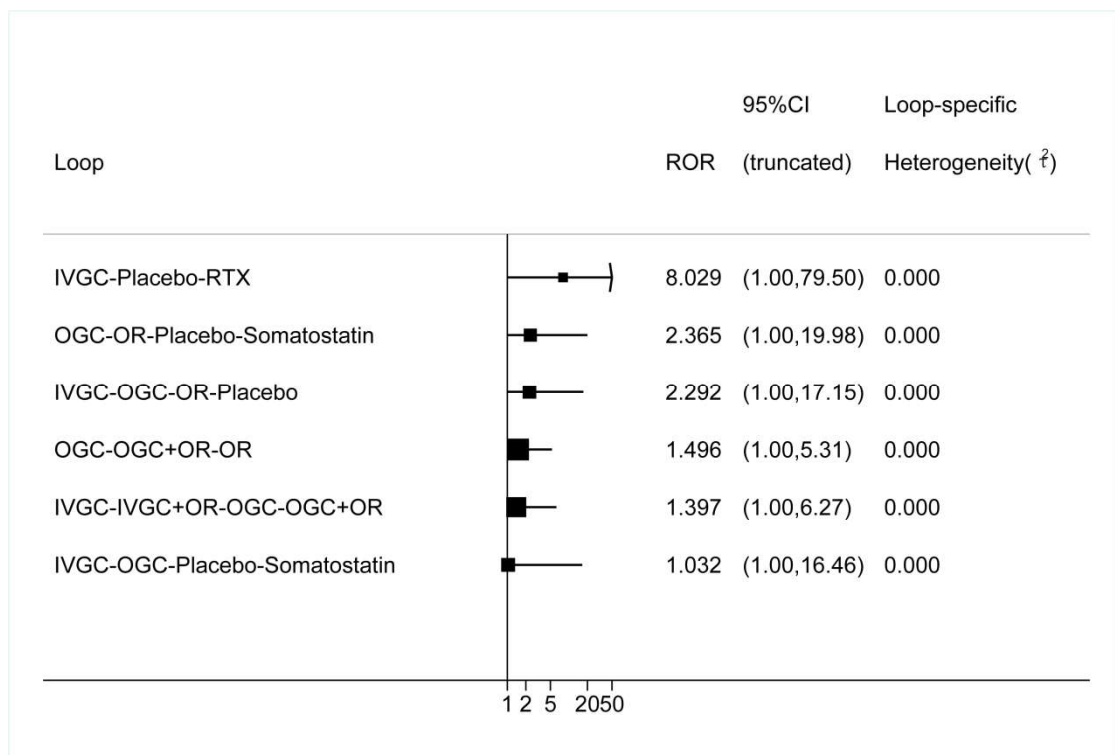

Fig. 2

Supplement: Supplementary Materials — The supplementary materials include two figures: Figure 1: risk of bias of the included RCTs. Figure 2: inconsistency test for direct and indirect comparison in the network meta-analysis. [file 7184163.f1.pdf]
